# Robust Parking Path Planning with Error-Adaptive Sampling under Perception Uncertainty

**DOI:** 10.3390/s20123560

**Published:** 2020-06-23

**Authors:** Seongjin Lee, Wonteak Lim, Myoungho Sunwoo

**Affiliations:** Department of Automotive Engineering, Hanyang University, 222 Wangsimni-ro, Seongdong-gu, Seoul 04763, Korea; seongjin90@gmail.com (S.L.); lwt1849@gmail.com (W.L.)

**Keywords:** automated parking, planning under uncertainty, replanning, utility theory

## Abstract

In automated parking systems, a path planner generates a path to reach the vacant parking space detected by a perception system. To generate a safe parking path, accurate detection performance is required. However, the perception system always includes perception uncertainty, such as detection errors due to sensor noise and imperfect algorithms. If the parking path planner generates the parking path under uncertainty, problems may arise that cause the vehicle to collide due to the automated parking system. To avoid these problems, it is a challenging problem to generate the parking path from the erroneous parking space. To solve this conundrum, it is important to estimate the perception uncertainty and adapt the detection error in the planning process. This paper proposes a robust parking path planning that combines an error-adaptive sampling of generating possible path candidates with a utility-based method of making an optimal decision under uncertainty. By integrating the sampling-based method and the utility-based method, the proposed algorithm continuously generates an adaptable path considering the detection errors. As a result, the proposed algorithm ensures that the vehicle is safely located in the true position and orientation of the parking space under perception uncertainty.

## 1. Introduction

Automotive industries have increasingly provided drivers with a variety of functions for safety and convenience. Automated parking systems that park autonomously without driver intervention are able to save time and reduce the risk of accidents. The process of a parking system starts with the detection of parking spaces using Around View Monitoring (AVM) systems [1,2,3,4] or ultrasonic sensors [5,6,7,8]. Then, based on the detected parking space, the parking system generates a parking path that leads the vehicle to the parking space safely. The system ends with the vehicle following the generated parking path until arriving at the parking space. Parking space detection and parking path planning are both important technologies. This is because a safe parking path can be planned with the accurate position and orientation of the detected parking space. If there is a detection error in the parking space, the parking path might collide with obstacles.

However, parking path planning in real driving is a challenging problem. In real driving, accurate parking space detection is required, but there are three factors that disturb precise detection: sensor noise, poor environments and an immature perception algorithm. The sensor noise that is always included in sensors is unpredictable. Even if sensor noise is completely removed, the measurement itself may be poor. For example, due to strong light, the image of a parking space from the AVM sensor can be too bright to be useful. In such environments, the detection performance of perception algorithms may not be perfect. For this reason, the perception system always has detection errors. However, if the detection error is considered in the planning process, the parking system can ensure arrival at the actual parking space without failure. For example, if the system generates a new parking path when the parking fails, safety can be assured. Previous commercial automated parking systems stop the vehicle if it judges that there is an observation error in the parking space. Otherwise, the system might cause crashes into obstacles due to detection faults.

Over the last few years, some researchers have developed parking path planning algorithms that locate the vehicle in an error-free parking space. These parking path planning algorithms are divided into three types: geometric methods, graph-searching methods, and optimization methods. Geometric methods find a solution by combining geometric primitives, such as straight lines, circles, and spirals [9,10,11]. These methods are simple but only operated in a predefined situation. Graph-searching methods include Hybrid-A* or RRT [12,13,14]. These methods construct a graph from the environment, then find an optimal path in the graph. While these methods can generate a parking path in a variety of environments, the main disadvantage of these approaches is that the execution time increases exponentially as the graph becomes more complex in a narrow environment. Optimization methods are divided into two methods; the first group [15,16,17] utilizes Model Predictive Control (MPC) to solve a global optimization problem, and the second group [18,19] uses the Optimal Control Problem (OCP) to solve a local optimization problem. MPC-based methods are almost able to find a solution wherever the parking space is; however they can take over one hundred seconds to find a solution [16,17]. On the other hand, OCP-based methods solve the problem with local optimization with the advantage that the execution time is similar to geometric methods’, and find an optimal path regardless of the vehicle’s position. Especially, the method proposed by Zips et. al. [18], has higher probability of finding a solution than Hybrid-A* which is considered as the state-of-the-art in Monte Carlo simulation. However, all algorithms including the OCP-based method are planned in a fully known environment. In other words, these algorithms cannot directly be used in real-driving, since they cannot consider the perception error from sensors. To reflect the perception error into such algorithms, additional methods for perception uncertainty are required.

From the perspective of perception uncertainty, there are probabilistic sampling methods and utility-based sampling methods for environments where perception errors exist. Some researchers [20,21,22,23,24] represent external uncertainty as the probability of the ego position and then sample nodes in the probability boundaries. On the other hand, other researchers [25,26,27,28] propose motion planning algorithms that consider environmental uncertainty as the probability of obstacle boundaries, then sample nodes by avoiding the obstacle boundaries. These algorithms sample a node of the searching-tree avoiding the uncertainty that the parking space has an error. In other words, these methods cannot explicitly express the uncertainty of the node. In addition, these methods take a long time to execute in narrow environments since they require an enormous number of sample nodes to find a goal in such environments. On the other hand, utility-based sampling methods are proposed to reflect perception uncertainty directly in the planning process [29,30,31]. Utility-based sampling methods explicitly express the uncertainty by directly representing the preference of the sample node. The advantage of utility-based sampling methods is that their expression is scalable from sample nodes to sample paths. For example, by formulating a user-defined utility function, both the preference and the uncertainty of each path can be estimated to select the best path. If the OCP-based method, which has the highest probability of finding a parking path, and the utility-based sampling method, which has scalability to paths, are combined, not only can the perception error in the real world be effectively considered but the execution time can also be short.

As explained so far, the OCP-based optimization method has the advantage of generating a parking path in narrow environments within a short execution time, and utility-based sampling methods have advantages of uncertainty expression from the perspective of re-planning. Therefore, if we generate path candidates through OCP-based optimization methods, then consider the uncertainty of parking spaces using utility-based sampling methods, automated parking systems can locate the vehicle in an actual parking space with a small error even if there is an error at the parking space. In this paper, we propose a parking path planning algorithm that recursively selects an optimal path by considering the utility of each path among the path candidates generated using the OCP-based optimization method. Since the proposed algorithm directly reflects the uncertainty of the parking space in the planning process, the algorithm helps to accurately locate to the actual parking space. In addition, if the vehicle is regarded as not reaching the actual parking space, the proposed algorithm re-generates the optimal path.

The remainder of this paper is arranged as follows. The overview of the proposed method is provided in Section 2. Then, the proposed method is divided into three parts: Parking Space Sampling, Path Candidate Generation, and Optimal Path Selection. These are explained from Section 3, Section 4, Section 5. The experimental results are shown in Section 6, and this paper is concluded in Section 7.

## 2. Overview

**Figure 1:**
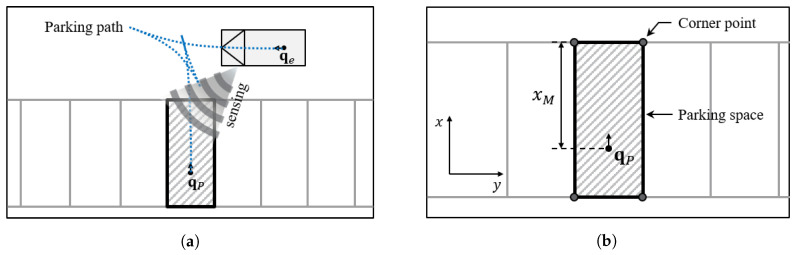
The overall process of parking path planning (**a**), and the definition of a parking space and corner points (**b**).

The overall process of parking path planning is described in Figure 1a. First, the vehicle, which is located in $q_{e}$, tries to start from outside of the parking space. At that time, the parking space is defined as a space consisting of four corner points as shown in Figure 1b. The target state $q_{P}=\left(x_{P},y_{P},\theta_{P}\right)$ indicates position and orientation of the parking space where the center of the rear wheel of the vehicle ($q_{e}$) should be at this location. The distance between the front line and $q_{P}$, which is represented $x_{M}$, depends on the vehicle length.

The target state $q_{P}$ is estimated from the parking space detected by sensors. However, due to the perceptional error, the target state can be incorrectly estimated. The inaccurate target state causes the parking path to lead the vehicle out of the parking space. For this reason, we propose a robust parking path generation algorithm to move the vehicle accurately to the target state.

**Figure 2:**
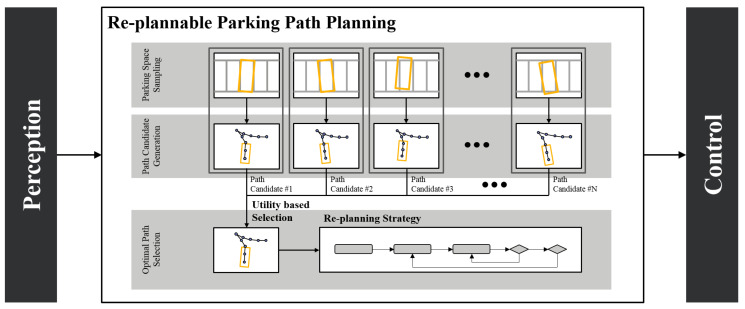
The overall architecture of re-plannable parking path planning: parking space sampling, path candidate generation, and optimal path selection.

The proposed method is shownin Figure 2. The whole process is divided linto three steps: *Parking Space Sampling*, *Path Candidate Generation*, and *Optimal Path Selection*. In the first step, we sample the target states where the actual parking space may be using the perceptional error model. Then, the OCP-based algorithm is employed to generate parking paths for each sampled target state. Finally, we calculate the utility of each path candidate. During this process, the planner decides whether to re-plan by considering the uncertainty of the target state. Each step is described in detail in the following sections.

## 3. Parking Space Sampling

The precise position and orientation of the parking space, which is called a target state, is important for parking path planning. There are free space-based approaches [5,6,7,8] and separate line-based approaches [1,2,3,4] for recognizing the parking space. Free space-based approaches search for vacant space between obstacles. On the other hand, the separate line-based approaches find the vertical and horizontal lines of the parking slot. Then, these approaches find the corner points of the parking space from each vacant space or separate lines. For the precise detection of the target state, the position of the corner points should be accurate. However, due to sensor noise, the natural vibration of the vehicle, and imperfection of the perception algorithms, the position error of the detected corner points increase. This effect cannot be ignored in the parking path planning. Parking can easily fail if the parking planner uses the parking space generated from the erroneous corner points. To reduce the effect of the erroneous corner points in the planning process, we propose the perceptional error model. By using the perceptional error model, the corner points from the perception system are sampled. Then, the planner generates parking paths from each sampled target state using the sampled corner points. Through this process, the effect of the erroneous corner points can be minimized.

### 3.1. Perceptional Error Model

The perceptional error starts from sensor noise and increases through the perception algorithm. Since perceptional errors are distributed differently according to the sensor type and algorithm, a different perceptional error model is required depending on the type. However, the objective of this paper is not to evaluate the quality of this model but whether our planner would work with a wide variety of models. We believe that the perception error model used for our experiments is representative and captures the important characteristics of frequently used perception modalities.

Many researchers have studied parking space detection algorithm using various sensors, such as ultrasonic sensors [5,6,7,8], AVM systems [1,2,3,4], and Lidar [32,33]. The AVM system is widely used for parking space detection since the AVM sensor can detect empty parking spaces, unlike ultrasonic sensors that can only search for empty spaces between two vehicles and has the advantage of being cheaper than Lidars. The AVM system calculates the location of a parking space from the image by applying the geometric relationship between the camera pose and the road surface.

**Figure 3:**
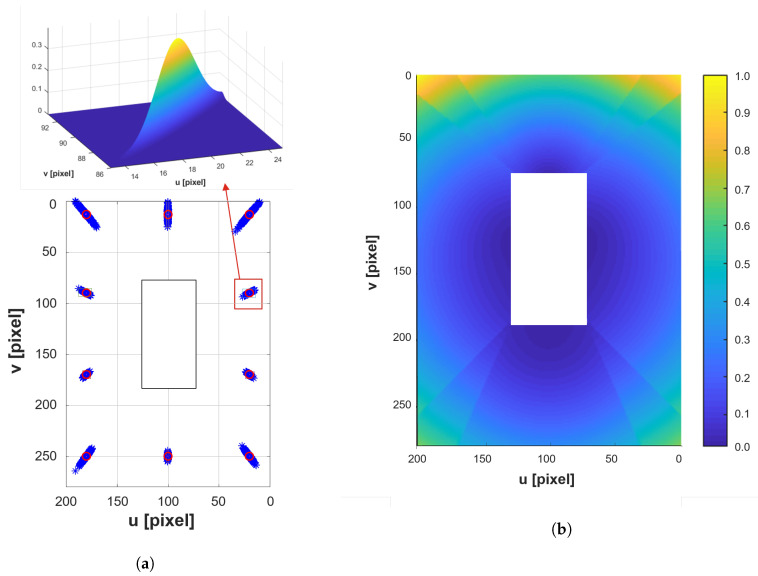
Variance of the Gaussian noise distribution for the AVM system (**b**) due to the vehicle motion caused by roll and pitch (**a**).

During parking, when vehicle motion (pitch and roll) occurs, the geometric relationship can be affected by the motion, which will cause measurement error. The change of the geometric relationship should be adjusted for reducing the measurement error. However, it is difficult to compensate for the change in the geometric relationship since it is not easy to measure the roll and pitch motion of the vehicle. Due to this phenomenon, the corner points detected by the AVM includes position errors as shown in Figure 3a. The fixed points, which is represented as the red dot, are measured as the blue dots when the vehicle is moving. For simplification, the measured points are modeled as Gaussian distribution at each pixel as shown in Gauss distribution in Figure 3a, and this can be expanded at every pixel like Figure 3b from [34]. In the measurement model of the AVM features, the variances tend to increase with farther distance from the ego vehicle. For example, the farther detected distance from the sensor has higher covariance and the shorter detected distance has lower covariance.

The perceptional error model is estimated from the sensor noise distribution. Contrary to the AVM noise distribution, the perceptional probability that the true position is located in the pixel decreases as the detected distance increases. This is conducted using the cumulative distribution function (CDF) of the Gaussian as follows:

(1) $$P_{detect}\left(d\right)=1-\frac{1}{2}\left(1+erf\left(\frac{d}{\sigma \sqrt{2}}\right)\right)$$

where “erf” is the Gauss error function and *d* is the detected distance from the nearest camera sensor. σ represents a tuning factor that controls the rate of the probability reduction of the detected distance. (1) represents an approximation of the true probability of the detected distance. Then, to apply the degradation from the perception algorithm, (1) is multiplied by the detection rate of algorithm λ.

(2) $$P_{perception}\left(d\right)=\lambda \cdot P_{detect}\left(d\right)$$

**Figure 4:**
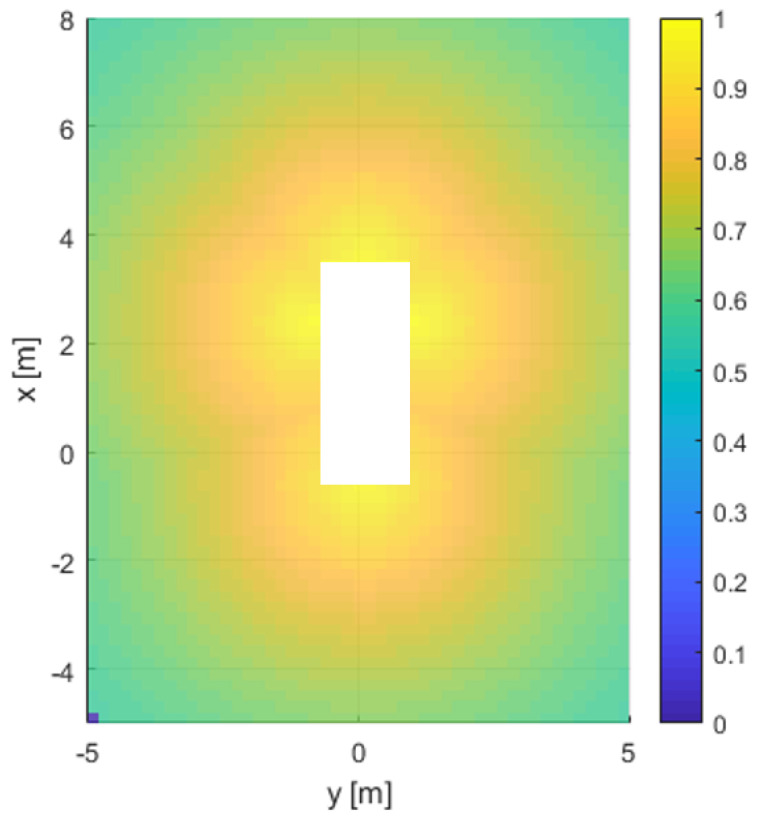
The perceptional error model from an AVM system to an alogrithm.

The perception algorithm can recognize and localize the corner points of the parking space, then estimate the target state from the detected corner points. Therefore, $P_{perception}\left(d\right)$ is the perceptional probability for the corner points of the parking space. The distribution of the perceptional probability of our system is shown in Figure 4. The perceptional probability becomes small as the distance from the sensor increases.

### 3.2. Error-Adaptive Sampling

**Figure 5:**
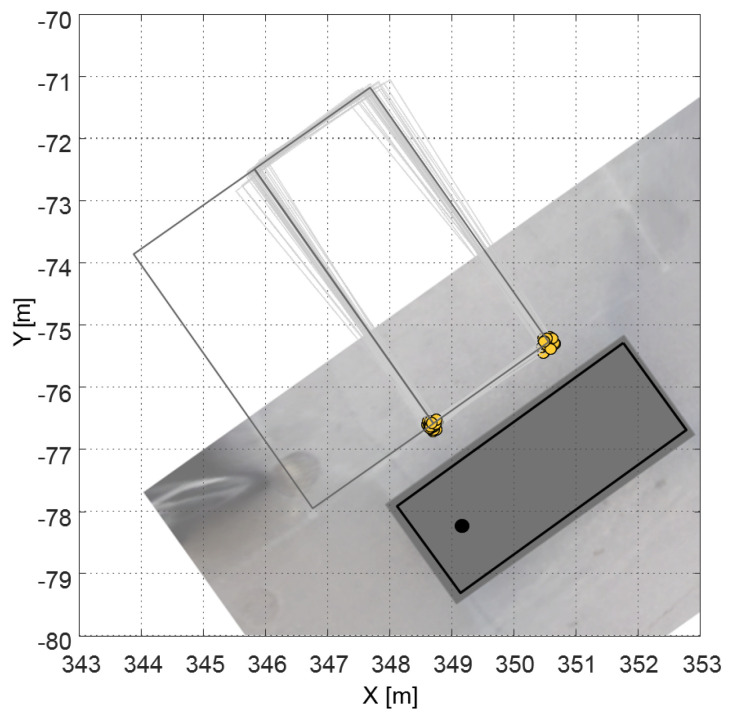
The parking spaces are generated through the sampled corner points; the yellow dots are sampled corner points, and the gray rectangles are the generated parking spaces.

As mentioned earlier in this section, we sample the corner points to represent the uncertainty of the perception system as shown in Figure 5. The sampled corner points (the yellow dots in Figure 5) are sampled according to the perceptional probability of the corner points. The target state and the probability of the target state are then calculated using the sampled corner points. We construct the whole parking space by using the sampled two corner points as the left and right corners of the parking space, as shown in the gray rectangles in Figure 5. When configuring the parking space from the corner points, the length of the parking space is from the regulations. Then, to provide the target state to *path candidate generation*, the target state is estimated by considering the length of the vehicle. During this sampling process, we assume that the sampled corner points had become normally distributed. This is achieved using the Gaussian distribution function:

(3) $$\exp\left(-\frac{1}{2}{\left(X-\hat{X}\right)}^{T}\Sigma^{-1}\left(X-\hat{X}\right)\right)$$

where X=(x,y) denotes the position of the corner points in Cartesian coordinates, $\hat{X}$ refers to the detected corner points from the perception algorithm, and Σ indicates the standard deviation depending on the detection probability in Section 3.1. If the sampled corner points are assumed to be normal and binomial, the standard deviation can be formulated by using the normal approximation of the binomial as follows:

(4) Σ=N·P·(1−P)

where *N* denotes the total number of samples. From (4), a large *N* makes the standard deviation large, which allows for a wider range of errors to be considered. However, the execution time increases since the number of paths that need to be generated increases. On the other hand, a small *N* ensures real-time execution, although only a narrow range of errors can be considered. In this paper, N=50 is used to sample the parking spaces.

## 4. Path Candidate Generation

Since we utilize 50 samples of target states, a path candidate should be generated with a high success rate of path generation in a few milliseconds to apply the real-time application. In the comparison result in [18], the OCP-based method has the highest success rate in Monte Carlo simulation and takes less than 20 ms while Hybrid-A*, considered the state of the art, operates within 150 to 200 ms. That is the reason why the OCP-based method is used to *Path Candidate Generation*.

To generate a set of path candidates to reach each sampled parking space, the OCP-based method [18] is iteratively operated for each sampled target state. By applying this method, only one path can be optimized to achieve the sampled target state. The OCP-based method is formulated with the kinematic model of the vehicle and a cost function based on a pose error between the ego vehicle and the target state.

### 4.1. Vehicle Kinematic Model

**Figure 6:**
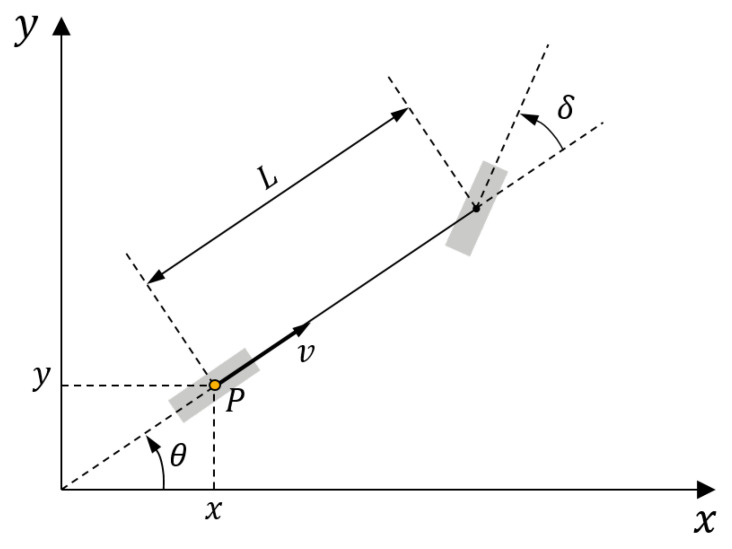
Vehicle kinematic model.

In the following, the vehicle model, as shown in Figure 6, depicts the mathematical description. Since the velocity of the vehicle is not over 10 kilometers per hour during parking, the tire slip angle is neglected. Therefore the vehicle can be simplified with the bicycle model, which has one front and one rear wheel. The motion of the vehicle is characterized by the coordinates (x,y) of reference point *P*, which is located at the center of the rear axle, orientation θ of the longitudinal axis, velocity *v* and steering angle δ. The differential equation of the vehicle model can be written as

(5) $$\frac{d}{dt}\left(\begin{array}{c} x\\ y\\ \theta \\ v\\ \delta \end{array}\right)=\left(\begin{array}{c} v\cos\theta \\ v\sin\theta \\ v\frac{\tan\delta }{L}\\ a\\ \psi \end{array}\right).$$

Here, $u_{A}={\left(a,\psi \right)}^{T}$ denotes the control input comprised of acceleration *a* and the steering angle rate ψ, and the parameter *L* refers to the wheelbase. For computational efficiency, this kinematic model can be decoupled by Path-Velocity-Decomposition [35]. With this decomposition, the velocity can be written as $v=D\frac{ds}{dt}$, with the path length *s* and the direction of the vehicle D∈{−1,1} (i.e., forward or backward). The kinematic state of the vehicle $q={\left(x,y,\theta \right)}^{T}$ can be obtained from (5), and the decoupled equation reads as follows:

(6) $$q^{{}^{\prime }}=\left(\begin{array}{c} x^{{}^{\prime }}\\ y^{{}^{\prime }}\\ \theta^{{}^{\prime }} \end{array}\right)=\left(\begin{array}{c} D\cos\theta \\ D\sin\theta \\ Du_{l} \end{array}\right)=f_{s}\left(q,u_{l},D\right)$$

(7) $$\frac{d}{dt}\left(\begin{array}{c} v\\ \delta \end{array}\right)=\left(\begin{array}{c} a\\ \psi \end{array}\right)$$

where ${\left(\cdot \right)}^{{}^{\prime }}$ denotes the derivative with respect to the path length *s*, and the new control input $u_{l}=\tan\delta /L$. To apply discrete optimization, (6) can be discretized with respect to the path length *s* by using second-order Runge-Kutta discretization

(8) $$k_{1_{i}}=\mu_{i}f_{s}\left(q_{i},u_{l_{i}}\right)$$

(9) $$k_{2_{i}}=\mu_{i}f_{s}\left(q_{i}+\frac{1}{2}k_{1_{i}},u_{l_{i}}\right)$$

(10) $$q_{i+1}=q_{i}+k_{2_{i}}+O\left(\mu_{i}^{3}\right)$$

with the step length $\eta \in [\eta_{min},\eta_{max}]$. Note that the step length is constrained by a minimum and maximum value, $\mu_{min}$ and $\mu_{max}$, respectively. Neglecting the error term $O(\mu_{i}^{3})$, the discretized equation reads as follows:

(11) $$\begin{array}{cc} q_{i+1}\; & =\left(\begin{array}{c} x_{i+1}\\ y_{i+1}\\ \theta_{i+1} \end{array}\right)=\left(\begin{array}{c} x_{i}+D\eta_{i}\cos\left(\theta_{i}+D\frac{\eta_{i}u_{l_{i}}}{2}\right)\\ y_{i}+D\eta_{i}\sin\left(\theta_{i}+D\frac{\eta_{i}u_{l_{i}}}{2}\right)\\ \theta_{i}+D\eta_{i}u_{l_{i}} \end{array}\right)\\ & =f(q_{i},u_{i},D) \end{array}$$

The new control input $u={\left(u_{l},\eta \right)}^{T}$ of (11) consists of the steering input $u_{l}\in \left[-0.3,0.3\right]\left[rad\right]$ and the step length η∈[0.1,0.5][m].

### 4.2. Parking Path Planning

Based on (11), a sequence of static optimization problems is formulated. The whole parking path is not calculated at once but only for one incremental step. Then, the optimization problem solves in a recursive manner with the calculation of a direction change point. The next subsection will explain how to change the direction. The one-step static optimization problem can be formulated as

(12) $$\begin{array}{cc} \underset{u_{i}}{\mathrm{argmin}}\; & l_{O_{i}}\left(q_{i+1}\right) \end{array}$$

(13) $$\begin{array}{cc} s.t.\; & q_{i+1}=f\left(q_{i},u_{i},D\right) \end{array}$$

(14) $$\begin{array}{cc} \; & h_{P}\left(q_{i+1}\right)\leq 0 \end{array}$$

(15) $$\begin{array}{cc} \; & u_{min}\leq u_{i}\leq u_{max} \end{array}$$

where the objective function (12) is expressed as,

(16) $$\begin{array}{c} l_{O_{i}}\left(q_{i}\right)=r_{\theta }e_{\theta_{i}}^{2}+e_{P_{i}}^{T}Re_{P_{i}}. \end{array}$$

Here, $e_{P_{i}}={\left(x_{i}-x_{target},y_{i}-y_{target}\right)}^{T}$ denotes the distance of the vehicle at iteration step *i* to the target position ${\left(x_{target},y_{target}\right)}^{T}$ and $e_{\theta_{i}}$ refers to the difference between the orientation of the vehicle $\theta_{i}$ and the orientation of the target orientation $\theta_{target}$. The weighting term R and $r_{\theta }$ with respect to each error should be positive definite and a positive value, respectively. There is one equality constraint and two inequality constraints. The first constraint in Equation (13) is equal to (11) for the vehicle kinematic constraint, as mentioned in Section 4.1. Then, the two inequality constraints in (14) and (15) are for the collision and control inputs, respectively. The collision constraint can be formulated with the Minkowski sum [36], and the control input constraint should be within the physical limits of the vehicle, which are the maximum steering angle and the maximum step length in one optimization iteration. Sequential quadratic programming (SQP) is used for solving optimization problem (12) to (15). SQP is one of the most successful methods for the numerical solution of constrained nonlinear optimization problems. SQP is an iterative procedure which models the nonlinear problem (NLP) for a given iterate by a quadratic programming (QP) subproblem, solves that QP subproblem, and then uses the solution to construct a new iterate. This construction is done in such a way that the sequence converges to a local minimum.

**Figure 7:**
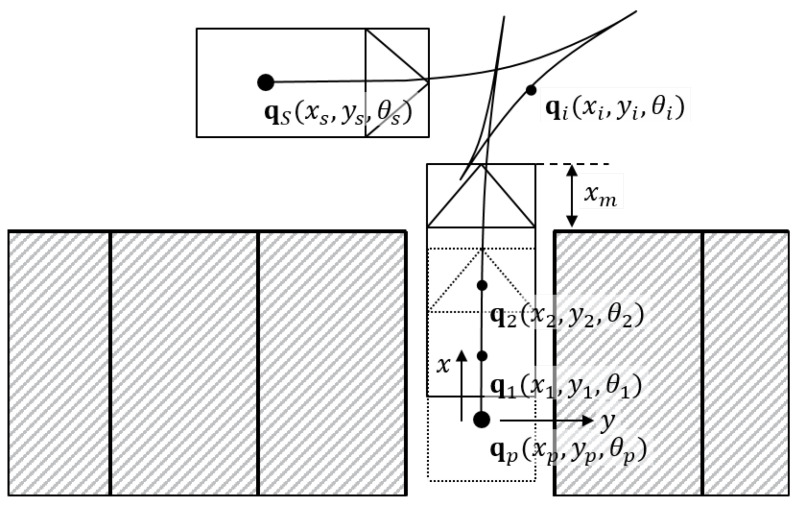
OCP-based parking path planning algorithm.

In every iteration step, the position and orientation of the vehicle are improved with respect to the cost function (16). In Figure 7, $q_{p}$ refers the target state of the parking space from Section 3 and $q_{s}$ denotes the start position and orientation of the ego vehicle. The state of each iteration step $q_{i}$ starts from $q_{p}$ and ends when $q_{i}$ reaches $q_{s}$. The optimization problem is only for one optimization step; this problem might be stuck in local minima. To solve the local minima problem, the algorithm is separated into two phases (A and B). During the phase A, the ego vehicle tries to escape from the parking space. The easiest way to escape the parking space is moving straight forward without any steering angle. At this time, the target orientation $\theta_{traget}$ is set to zero for the straight movement. For the same reason, $r_{\theta }=1.0$ and R are set to 1.0 and 0, respectively. The phase A continues until the vehicle reaches $x_{m}$ in Figure 7. After leaving the parking space, the second phase B is a step to locate the ego vehicle to the start pose $q_{s}$ with a direction change strategy which is explained in the following subsection. The first step of the phase B is changing the target state. Since the objective of the phase B is to align in the start position and orientation, the target state should be set to the start state $q_{target}=q_{s}$. The allocation movement in the phase B is different depending on $r_{\theta }$ and R. For example, high $r_{\theta }$ value makes the vehicle rotate first, while high $R_{\left(1,1\right)}$ value firstly reduces the x-directional error and high $R_{\left(2,2\right)}$ affects to reduce the y-directional error. To decide the weighting terms, we consider the way human parking. For example, the human driver firstly moves forward until passing the parking space in the y-direction. Then, he rotates the vehicle in the diagonal direction of the parking space. Finally, the vehicle is aligned in the x-direction and orientation of the parking space. From this, we can expect that the order of the important weighting terms is the y-direction ($R_{\left(2,2\right)}$), the orientation ($r_{\theta }$) and the x-direction ($R_{\left(1,1\right)}$). However, we find the path from the parking space $q_{p}$ to the start pose $q_{s}$ so that the order should be reversed. In this paper, we select $r_{\theta }=3.5$ and R=diag(25,1), respectively.

### 4.3. Direction Switching Point

To find a path before arriving at the starting position, the vehicle may exhibit changes in direction. There are two heuristic rules for directional change. The first rule is switching direction when the vehicle cannot move in that direction as shown in Figure 7. This means that the vehicle will collide with obstacles. The second rule is changing the direction when the current result of optimization $l_{O_{i}^{*}}$ is larger than the previous result $l_{O_{i-1}^{*}}$, as shown in Figure 7. The larger value of the cost function refers to a more distant position from the starting position. Therefore, the vehicle changes its direction to reduce the distance and orientation error from the starting position.

### 4.4. Obstacles

To generate a collision-free path, obstacles need to be expressed as constraints. If obstacles can be considered convex polygons, the computation time for optimization will be reduced. Therefore, obstacles are regarded as convex polygons. If the obstacle is non-convex, the obstacle was divided into a set of convex polygons through convex decomposition [36]. All convex polygonal obstacles are described as larger than their original size. During optimization, obstacles are transformed into inequality constraints in the cost function. The inequality constraints check whether the vehicle is within the obstacle boundary.

## 5. Optimal Path Selection

After generating path candidates, the planner should decide the optimal path that is likely to reach the true target state. This selection process does not work only once, but continues until the vehicle reaches the target state. However, if the path is continuously changed by the optimal selection, this path loses consistency with the previous path. That is why the re-planning strategy is required. In this section, we discuss the optimal selection and re-planning strategy.

### 5.1. Utility-Based Decision

**Figure 8:**
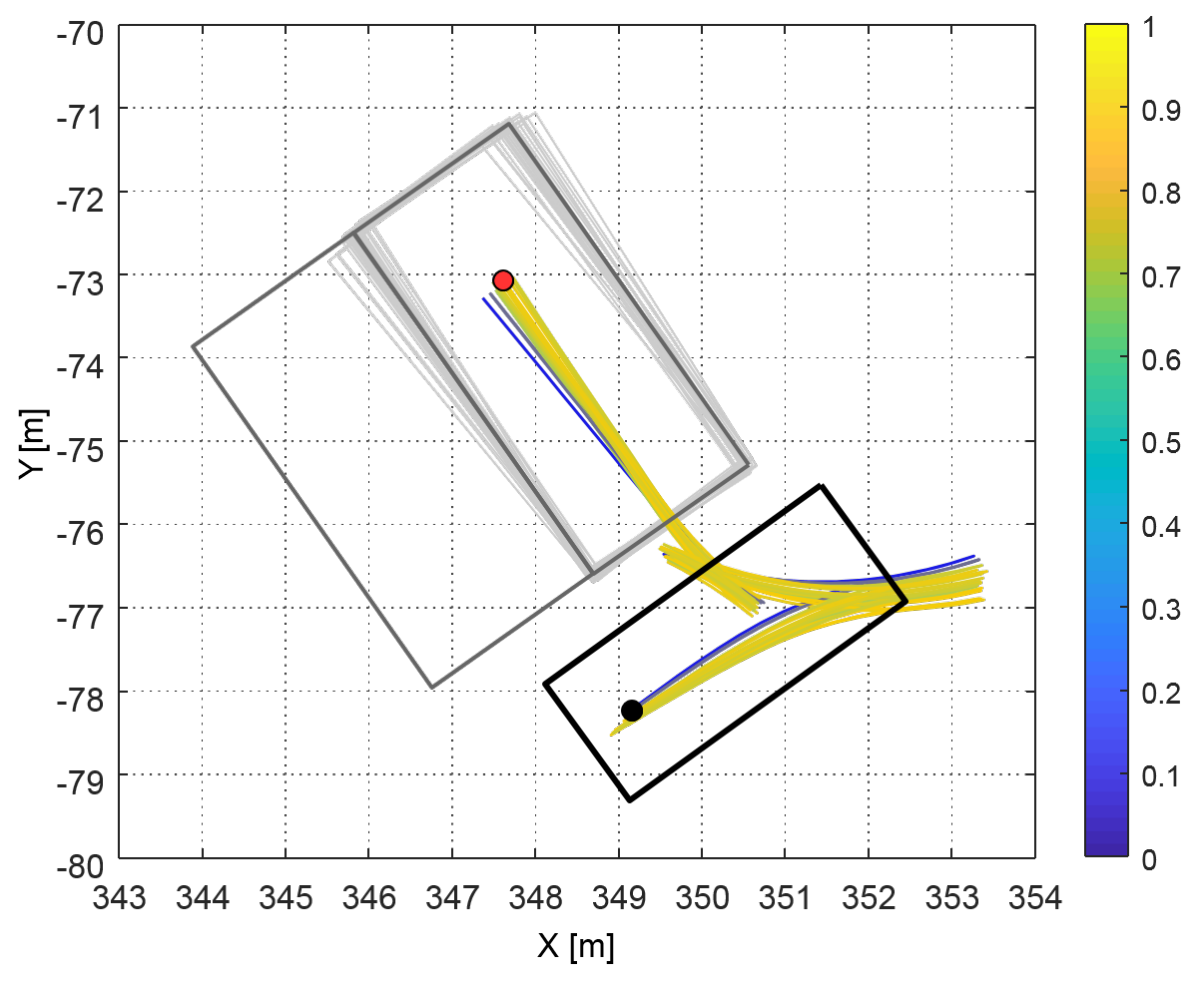
The utility of each path candidate is estimated; the path that is the nearest to the actual target state has the maximum expected utility.

This section focuses on how to make rational decisions based on the perceptional error model and utility function. This section begins by introducing the foundations of utility theory [37,38] and showing how it forms the basis for rational decision making under uncertainty. Based on utility theory, the degree of desirability with respect to two different statements can be compared and measured. The real-valued degree of desirability is called *utility*, and the utility of path *s* can be written as U(s). The utility is composed of a function of various task-specific considerations. The expected utility integrates the notions of utilities, weighted by the probability that the path is successful. This probability is consistent with the probability of the sampled target state in Section 3. The expected utility EU of path *s* is written as

(17) $$EU\left(s\right)=P\left(s\right)\cdot U_{ideal}\left(s\right)+\left(1-P\left(s\right)\right)\cdot U_{real}\left(s\right)$$

where $U_{ideal}\left(\cdot \right)$ measures how close the path candidate is to the detected target state. Through $U_{ideal}$, the ideal path to the actual target state is selected. $U_{real}\left(\cdot \right)$ estimates how near the path candidate is to the ego vehicle. The high value of $U_{real}$ means that the realistic path near the ego vehicle would be selected. P(·) measures the probability of the path candidate. The probability of the path candidate is equal to the probability of the sampled target state because only one path candidate can be generated from one target state. Based on (17), the probability of the path candidate determines whether the path is ideal or realistic. For example, if there is an ideal path candidate that has a high probability close to the detected target state, the vehicle follows the ideal path candidate. On the other hand, if all path candidates have low probability, the vehicle selects the realistic path candidate near the ego vehicle. The expected utility of path candidates is shown in Figure 8. The expected utility is higher when the path candidate is close to the target state and near the ego vehicle. Each of terms in (17) is covered in detail in the following section.

#### 5.1.1. Ideal Utility Function

The desirable path for achieving the goal is the path nearest to the currently detected target state. Even though there is an error in the currently detected target state, we assume that the perceptional system gives the most reliable information from insufficient information. To formulate the degree of nearness, a new parameter, $d_{ideal}$, which is the weighted summation of the position difference and the orientation difference between the end point of the path candidate and the sampled target state is formulated as

(18) $$d_{ideal}=e_{d}^{T}Q_{d}e_{d}^{T}$$

where $e_{d}=\left(x_{s}-x_{d},y_{s}-y_{d},\psi_{s}-\psi_{d}\right)$ denotes the position difference and the orientation difference in Cartesian coordinates, then $\left(x_{s},y_{s},\psi_{s}\right)$ is the position and orientation of the end point of the path candidate. Likewise, $\left(x_{d},y_{d},\psi_{d}\right)$ indicates the position and orientation of the current detected target state. $Q_{d}$ denotes the positive definite matrix for weighting each parameter and is a 3 × 3 matrix equal to $diag(w_{x_{d}},w_{y_{d}},w_{\psi_{d}})$. When $w_{x_{d}}$ and $w_{y_{d}}$ are high, the utility of the path candidate that has the end point near the target state is high. Likewise, when $w_{\psi_{d}}$ is high, the utility of the path candidate that arranges the vehicle accurately is high.

To define a normalized utility function, the maximum utility is assigned 1 and the worst utility is assigned 0. By using the Gauss error function (erf), the utility function can be normalized as

(19) $$U_{ideal}\left(s\right)=\frac{1}{2}\left(1-erf\left(\frac{d_{ideal}}{\sigma_{d}\sqrt{2}}\right)\right)$$

The maximum value is calculated at d=0 and the minimum value is calculated at $d_{ideal}=\infty$. $\sigma_{d}$ denotes the uncertainty of the perception system. This means the decreasing rate from $d_{ideal}=0$ to $d_{ideal}=\infty$. For example, since a small $\sigma_{d}$ reduces the utility even if it is slightly different from $d_{ideal}=0$, the perception system is regarded as certain. Conversely, a high $\sigma_{d}$ achieves similar utility around $d_{ideal}=0$.

#### 5.1.2. Realistic Utility Function

The reality of the path candidate is the degree to which the path candidate can be followed and punishes the collision path candidate. To check the physical collision, the Minkowski sum [36] is used. Since the output of the Minkowski sum is true or false, we use the Minkowski function by multiplying negative values to punish a collision. Then, similar to the ideal utility, the parameter $d_{real}$, which represents the closeness of the path candidate, is formulated as:

(20) $$d_{real}=e_{e}^{T}Q_{e}e_{e}^{T}$$

where $e_{e}=\left(x_{n}-x_{e},y_{n}-y_{e},\psi_{n}-\psi_{e}\right)$ refers to the position difference and orientation difference between the ego vehicle and the nearest path point. The subscript notations *e* and *n* represent the ego vehicle and the nearest path point, respectively. $Q_{e}$ denotes the positive definite matrix for weighting the error and is equal to $diag(w_{x_{e}},w_{y_{e}},w_{\psi_{e}})$. Then, the Gauss error function can also be used to normalize utility. The total realistic utility function is as follows,

(21) $$U_{real}\left(s\right)=\frac{1}{2}\left(1+erf\left(\frac{d_{real}}{\sigma_{e}\sqrt{2}}\right)\right)+\alpha \cdot f_{Minkowski}$$

where $f_{Minkowski}$ refers to the Minkowski function, which checks a collision, and α=−10,000 denotes the punishment factor for not selecting the collision path candidate.

### 5.2. Re-Planning Strategy

**Figure 9:**
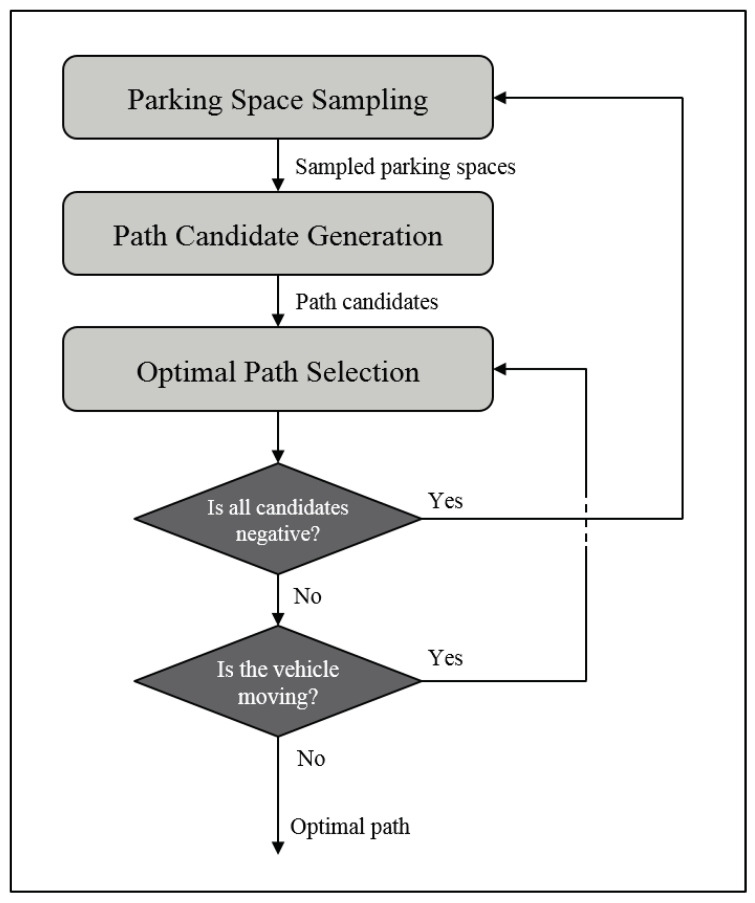
Flow diagram of the overall re-planning strategy.

The overall re-planning diagram is shown in Figure 9. The initial information might be insufficient for locating the vehicle in the actual parking space because of the uncertainty of the perception system. If the uncertainty is high or all paths are considered to collide, the maximum expected utility may be lower than the threshold utility $U_{threshold}$. In this case, it is regarded that there is no available path. Then, the planner’s only option is to return the *Parking Space Sampling*.

When the maximum expected utility is over $U_{threshold}$, the planner starts to park. However, due to the noisy measurement, if the planner selects the optimal path at every step, the vehicle loses its consistency. In other words, the vehicle will stagger. To achieve stability, the optimal path is only selected when the vehicle stops.

## 6. Experimental Results

**Figure 10:**
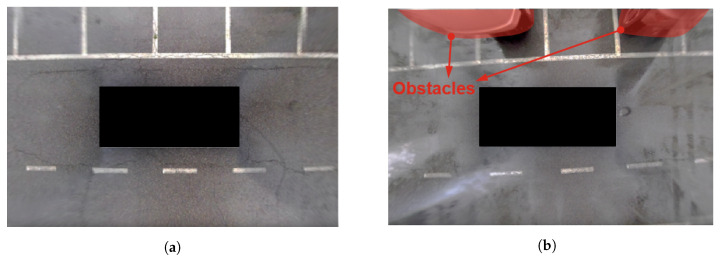
The AVM images of two test scenarios: open scenarios and occupied scenarios. (**a**) An open scenario; there is no other obstacles around the vehicle, (**b**) An occupied scenario; there are other obstacles around the vehicle.

We evaluated the proposed algorithm in two scenarios as shown in Figure 10. The first scenario was in the parking lot, where other obstacles did not exist. In the second scenario, the ego vehicle tried to park in a parking space where other vehicles were already parked on both sides of it. The evaluation shows how many errors the algorithm can accommodate during parking compared with the planner without re-planning, and the position and orientation error were calculated in the test scenarios.

**Figure 11:**
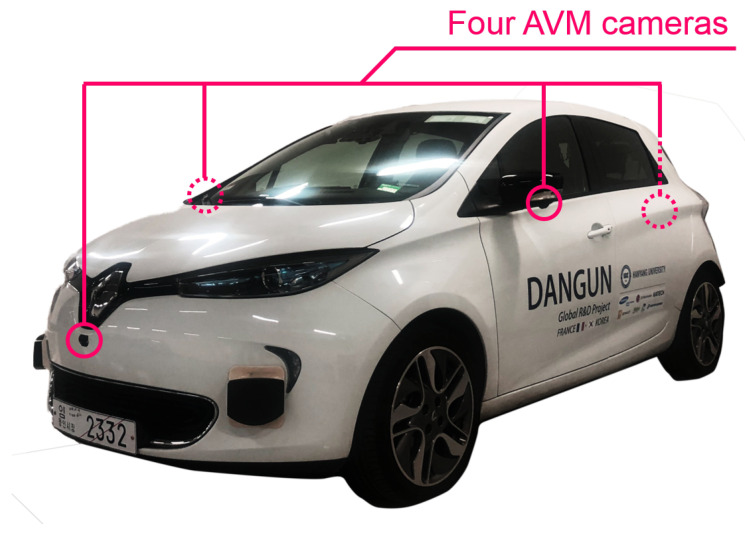
Test vehicle ZOE equipped with AVM and motion sensors.

The tests were conducted using the test vehicle ZOE from Hanyang University (Figure 11). The dimensions of the vehicle were as follows: the total length was 4.084 m, the total width was 1.730 m, and the wheelbase was 2.845 m. Then, we chose a parking lot with the dimensions 2.3 m × 5.0 m, as specified by the Department of Transportation. The surface of the parking lot was uneven, inclined or declined. Such parking lots might cause unexpected errors for a perception system. Nevertheless, we wanted to show that the proposed system completes parking in these conditions.

The test vehicle was equipped with AVM and wheel speed sensors. For parking space detection, the semantic segmentation-based algorithm from AVM images [39] was implemented. This algorithm finds the intersecting points between the vertical lines and horizontal lines from the semantic segmented lines of the parking spaces. The position of the vehicle was estimated by dead reckoning using a motion sensor [17,40,41]. Then, the vehicle was controlled by control inputs with Model Predictive Control (MPC) in the lateral [42,43,44,45] and longitudinal directions [46,47] separately.

The proposed planning algorithm was implemented in an i5 Core embedded PC in the C++ language with a Robot Operating System (ROS) environment [48]. The static optimisation problem is solved using the SQP-algorithm of the numeric software library NLopt [49,50]. The execution periods of parking space sampling, path candidate generation, and utility-guided sampling were 50 milliseconds, respectively. All the algorithms were operated in multiple threaded environments.

### 6.1. Scenario 1: Open Scenario

**Figure 12:**
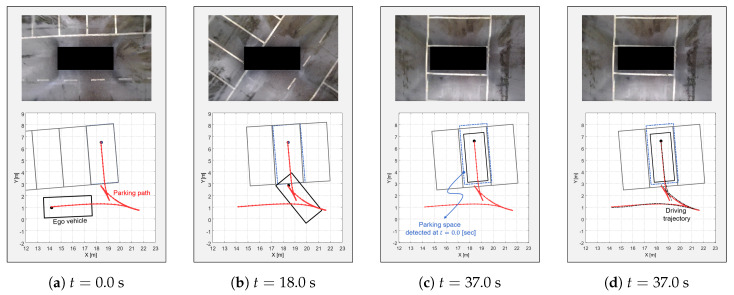
The open scenario without re-planning; there were position and orientation differences between the first (**a**) and last detected parking spaces (**c**).

The parking process in the open scenario (see Figure 10a) without a re-planning scheme is shown in Figure 12. In this case, the planner generated a parking path (the red line) using the first detected parking space (the blue dashed rectangle). The driving trajectory of the ego vehicle is shown as the black dashed line in Figure 12c. As a result, the vehicle was in the first detected parking space even though there was a detection error in this space. In this case, there were no obstacles around the vehicle; however, if other vehicles were already parked next to the ego vehicle, this position error might have caused an accident.

**Figure 13:**
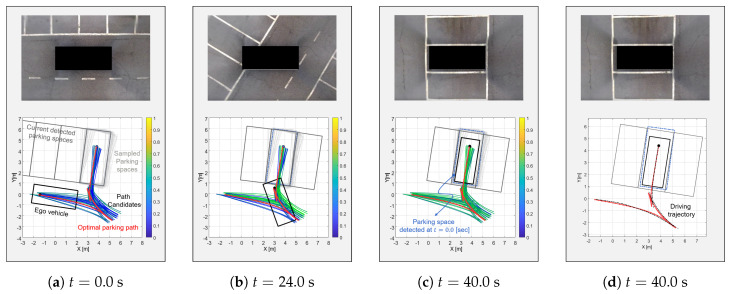
The open scenario with re-planning; there were also differences between the first and last detected parking spaces; however, the proposed algorithm changed the optimal path according to position and orientation differences.

On the other hand, Figure 13 shows the overall process of the proposed algorithm. Firstly, the sampled parking spaces were created such as the light gray rectangles in Figure 13 at t = 0 s. Then, each utility of the generated path candidates was calculated and described as spectrum lines. Through this process, the optimal path, which was considered a perception error, was selected at t=0 s (the red line). The vehicle started from the optimal path. Then, the detected parking spaces, which are represented as dark gray rectangles, continuously changed during parking. Due to the changed parking space, the vehicle followed another optimal path when the maximum expected utility was calculated again using (17). The position and orientation difference between the first detected parking space and the last detected parking space (the blue dashed rectangle and the gray rectangle in Figure 13c) are over 15 cm and 2.5 deg, respectively. This value can cause a crash if there is another vehicle next to the parking space. Through the proposed algorithm, the vehicle reached the actual parking space with a smaller error than the planner without re-planning. For understanding, the entire process of the re-planning is described in the Supplementary Materials.

### 6.2. Scenario 2: Occupied Scenario

**Figure 14:**
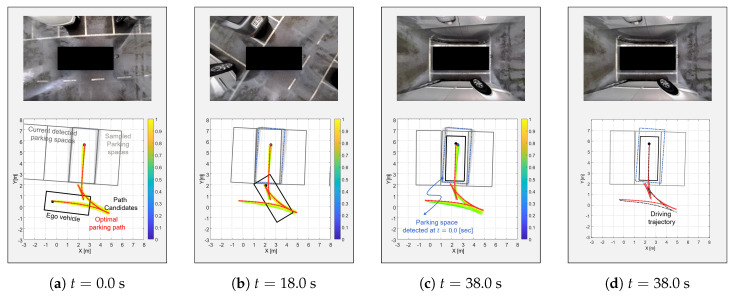
The occupied scenario with re-planning; the proposed algorithm detected the change of the parking space, then, changed its optimal path to locate the vehicle in the actual parking space.

The process of the proposed algorithm in the occupied scenario is shown in Figure 14. At t = 0.0 s, the vehicle tried to park in the parking space between two other vehicles. The proposed planner first generated the path candidates as shown by the spectrum lines in Figure 14. Unlike the open scenario, the utilities of the path candidates were higher than the open scenario case since the detected parking space was closer than the open scenario case. The closer parking space made the probability of the parking space high. This is why the spectrum lines appeared more yellow. In addition, the path candidate that was considered to be colliding was punished by Equation (21). Then, the proposed algorithm selected the optimal path (the red line) and started to follow it. At t = 18.0 s, the planner changed the optimal path since the change of the parking space was recognized and the previous optimal path collided with the other vehicle. Similarly, the proposed algorithm constantly changed the path each time it stopped, checking for collisions and changes in the parking space until it reached the parking space. As shown by the blue dashed rectangle of Figure 14c, the parking space was finally changed by over 20 cm and 5 deg in the x and y direction and orientation, respectively. If the vehicle had not changed its path, it would have collided with obstacles. Even though there was a change in the parking space, the proposed algorithm accommodated the perception error and then re-planned the optimal path in the erroneous parking space.

**Figure 15:**
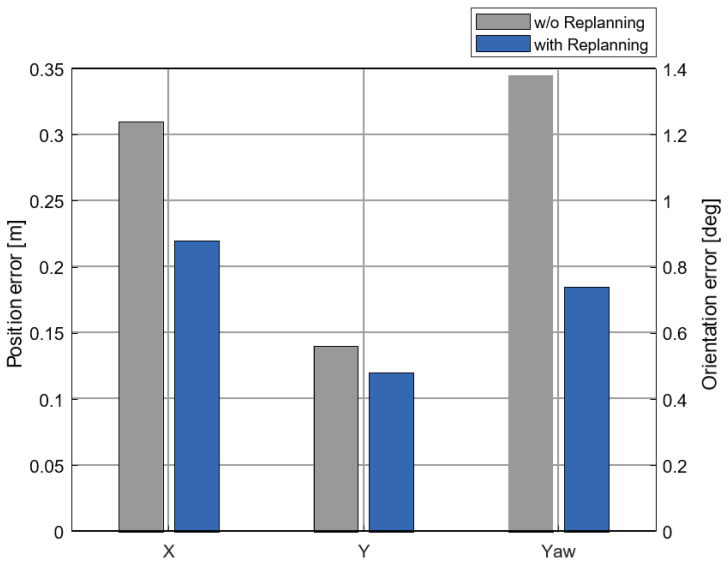
The comparison of average errors between the proposed algorithm and the planner without re-planning.

We evaluated each scenario 100 times to check the capability and robustness of the proposed algorithm. The results were verified by the average error of the position and orientation. As shown in Figure 15, the average error of the proposed algorithm was reduced by 27.6, 19.8, and 46.3 percent in each x and y direction and orientation. These reduced values prevented collisions with other vehicles in the occupied cases. In addition, the success rate of the proposed algorithm was increased from 86 to 93 percent compared to the planner without re-planning. At that time, parking was regarded as fail when some of the vehicle crossed the parking slot marker.

## 7. Conclusions

This paper presents adaptive parking path planning for uncertain environments. The proposed algorithm was comprised of three steps: *parking space sampling*, *path candidate generation*, and *optimal path selection*. To reflect the uncertainty of the perception system, a perceptional error model was proposed in the *parking space sampling* step. The perceptional error model estimated the probability of the parking space, which was the result of the perception system, to sample possible parking spaces. By applying optimization-based parking path generation, a number of parking path candidates were quickly generated to achieve the sampled goals in *path candidate generation*. Then, each utility of the path candidates was evaluated from the perspectives of reachability and consistency in the *optimal path selection* step. While the vehicle was parking, the utilities of each candidate were changed according to the detected parking space and the previous optimal path, then the new optimal path was selected to reach the actual parking space accurately.

The experimental results show the capability to continuously generate the adaptive paths for the perception errors. Through the proposed algorithm, the vehicle could reach an actual parking space even though the input of the planner, which was the currently detected parking space, was continuously changed. In such erroneous perception systems, the proposed algorithm could locate the vehicle in an actual parking space with fewer errors than the parking path planner that does not replan. Moreover, the feasibility of re-planning is shown when the vehicle is expected to collide. The extensive experiments prove that the proposed algorithm is able to provide the safest path among the possible parking path candidates.

Nevertheless, future work will be extended in four ways. On the first hand, the idea is to include various sensor models and detection algorithms which are not considered in this paper. Sensor model can be expanded to Radar and ultrasonic sensors which are widely used to the automated parking system. The second is to cover not only perpendicular parking but also parallel parking, angle parking, and forward parking. This can be covered in Path Candidate Generation part by changing the appropriate target position and orientation. The third approach may be extended by dealing with dynamic obstacles that can cause the change of the path. Finally, we expand the proposed method to automated valet parking system. To achieve the automated valet parking system, it is necessary to consider dynamic obstacles in areas that are blinded due to vehicles in the parking lot.

## Supplementary Materials

The following are available at https://www.mdpi.com/1424-8220/20/12/3560/s1, Video S1: Robust 442 Parking Path Planning with Error-Adaptive Sampling under Perception Uncertanity. A supporting video article is 443 available at doi: link.
